# Proline-Dependent Induction of Apoptosis in Oral Squamous Cell Carcinoma (OSCC)—The Effect of Celecoxib

**DOI:** 10.3390/cancers12010136

**Published:** 2020-01-06

**Authors:** Natalia Tołoczko-Iwaniuk, Dorota Dziemiańczyk-Pakieła, Katarzyna Celińska-Janowicz, Ilona Zaręba, Agnieszka Klupczyńska, Zenon J. Kokot, Beata Klaudia Nowaszewska, Joanna Reszeć, Jan Borys, Wojciech Miltyk

**Affiliations:** 1 Department of Maxillofacial and Plastic Surgery, Medical University of Bialystok, Sklodowskiej-Curie 24a, 15-276 Bialystok, Poland; beata.nowaszewska@wp.pl (B.K.N.); jan.borys@umb.edu.pl (J.B.); 2 Department of Otolaryngology, Provincial Hospital in Bialystok, Sklodowskiej-Curie 26, 15-278 Bialystok, Poland; dyrka2@wp.pl; 3 Department of Pharmaceutical Analysis, Medical University of Bialystok, Mickiewicza 2D, 15-522 Białystok, Poland; katarzyna.celinska-janowicz@umb.edu.pl (K.C.-J.); wojciech.miltyk@umb.edu.pl (W.M.); 4 Department of Medicinal Chemistry, Medical University of Bialystok, Mickiewicza 2D, 15-522 Białystok, Poland; ilona.zareba@gmail.com; 5 Chair and Department of Inorganic and Analytical Chemistry, Poznan University of Medical Sciences, Grunwaldzka 6, 60-780 Poznan, Poland; aklupczynska@ump.edu.pl (A.K.); zjk@ump.edu.pl (Z.J.K.); 6 Department of Medical Pathomorphology, Medical University of Bialystok, Waszyngtona 13, 15-269 Bialystok, Poland; joasia@umb.edu.pl

**Keywords:** oral cancer, oral squamous cell carcinoma, apoptosis, proline, proline dehydrogenase/oxidase, celecoxib

## Abstract

*Background*: Oral squamous cell carcinoma remains a significant worldwide public health challenge, associated with high morbidity and mortality. Treatment of this type of cancer lacks effective medication. Moreover, there are very few specific biomarkers that are useful in early diagnosis or treatment optimisation. Proline metabolism may prove to be of importance in the search for new treatment modalities. *Methods*: To evaluate the significance of proline metabolism in the development of oral cancer, proline concentration was assessed in oral cancer tissue and normal oral mucosa. The results were compared to the clinical stage and histological grade of the tumours. Moreover, the expression of proteins involved in proline metabolism via proline dehydrogenase/oxidase (PRODH/POX, PPARγ, HIF1-α) was determined. In the next stage of the study, conducted on cell lines of tongue cancer treated with celecoxib, the aforementioned factors involved in proline metabolism were evaluated. Cellular viability and cell proliferation, as well as apoptosis, were also assessed. *Results*: Our research results indicate that a high intracellular proline concentration and expression of factors involved in its metabolism correlate with the clinical stage and histological grade of oral cancer. Moreover, we are the first researchers to demonstrate that celecoxib can affect proline metabolism, causing an increase in pro-apoptotic factors (PRODH/POX, PPARγ), reducing the expression of HIF-1α and activating apoptosis. *Conclusions*: Proline metabolism, due to its involvement in the process of apoptosis, can be of great importance in anticancer therapy. It appears that celecoxib, which influences the PRODH/POX pathway, may be a promising therapeutic compound in oral cancer treatment.

## 1. Introduction

Oral squamous cell carcinoma (OSCC) represents 90% of all malignancies in the oral cavity. It remains a significant global public health challenge, associated with high morbidity and mortality [1]. According to the latest report on the global incidence of cancer (GLOBOCAN 2018), lip and oral cavity cancer ranks eighteenth by incidence, with 354,864 new cases and 177,384 deaths per year. It is worth noting that these numbers are increasing as illustrated by the comparison with the relevant data from 2012 (morbidity 300,373; mortality 145,353) and are predicted to rise further (by 62% by the year 2035). Despite significant advances in the diagnosis and treatment of this disease, the overall survival rate has not changed and is estimated at approximately 50% [2,3,4,5]. Among the available treatment methods, surgical resection and radiotherapy are recommended. Deformities and functional deficits caused by the development of malignant tumours in this location as well as by the treatment modalities mentioned above often produce profoundly negative psychological and social effects. They usually impede major life activities such as breathing, nutrition, speech, vision or smell [6,7]. The situation is compounded by the fact that there are very few specific biomarkers useful in early diagnosis which could help predict malignant transformation and optimise therapy. Therefore, a search for the solution to this problem constitutes an important direction in the development of biomedical sciences [1,8].

An important factor which may have an impact on cancer development is proline metabolism. Proline (Pro) is an endogenous amino acid which plays a significant role not only in protein biosynthesis but is also a key regulator of many biochemical and physiological cellular processes such as oxidative stress, immune response or intercellular signalling. Proline also serves as a basic amino acid for the synthesis of polyamines (key regulators of DNA biosynthesis, cell proliferation and differentiation) [9,10]. Reports in the available literature indicate that high proline concentration in cancer cells (particularly oesophageal and pancreatic cancer) is associated with poor histological differentiation of cells and a high clinical stage of malignancy [11,12]. An important enzyme in the process of carcinogenesis, which catalyses the first step of proline degradation, is proline dehydrogenase/oxidase (PRODH/POX). It catalyses the oxidation of proline to Δ1-pyrroline-5-carboxylic acid (P5C). During this process, reactive oxygen species (ROS) and ATP are generated. It has been proven that PRODH/POX can induce apoptosis both via an extrinsic and intrinsic pathway [13,14]. Studies have also demonstrated a relationship between proline oxidase and cyclooxygenase II activity (COX-2), where the high expression of PRODH/POX inhibited COX-2 expression [15]. COX-2 overexpression has been revealed in cancerous tissue, which has also correlated with a worse prognosis in several types of malignant neoplasms [16]. The anticancerous potential of celecoxib, a COX-2 inhibitor, has been proven in many studies, mainly in colon, breast and prostate cancer as well as precancerous lesions, such as familial intestinal adenomatous polyposis and Barrett’s oesophagus [17,18]. However, its precise mechanism of activity in this area remains unclear [19].

The purpose of the study was to evaluate the role of proline metabolism in the apoptosis of oral cancer and the impact of celecoxib on this process.

## 2. Results

### 2.1. Proline Concentration in Oral Cancer Tissue in Comparison to Normal Oral Mucosa

**Figure 1:**
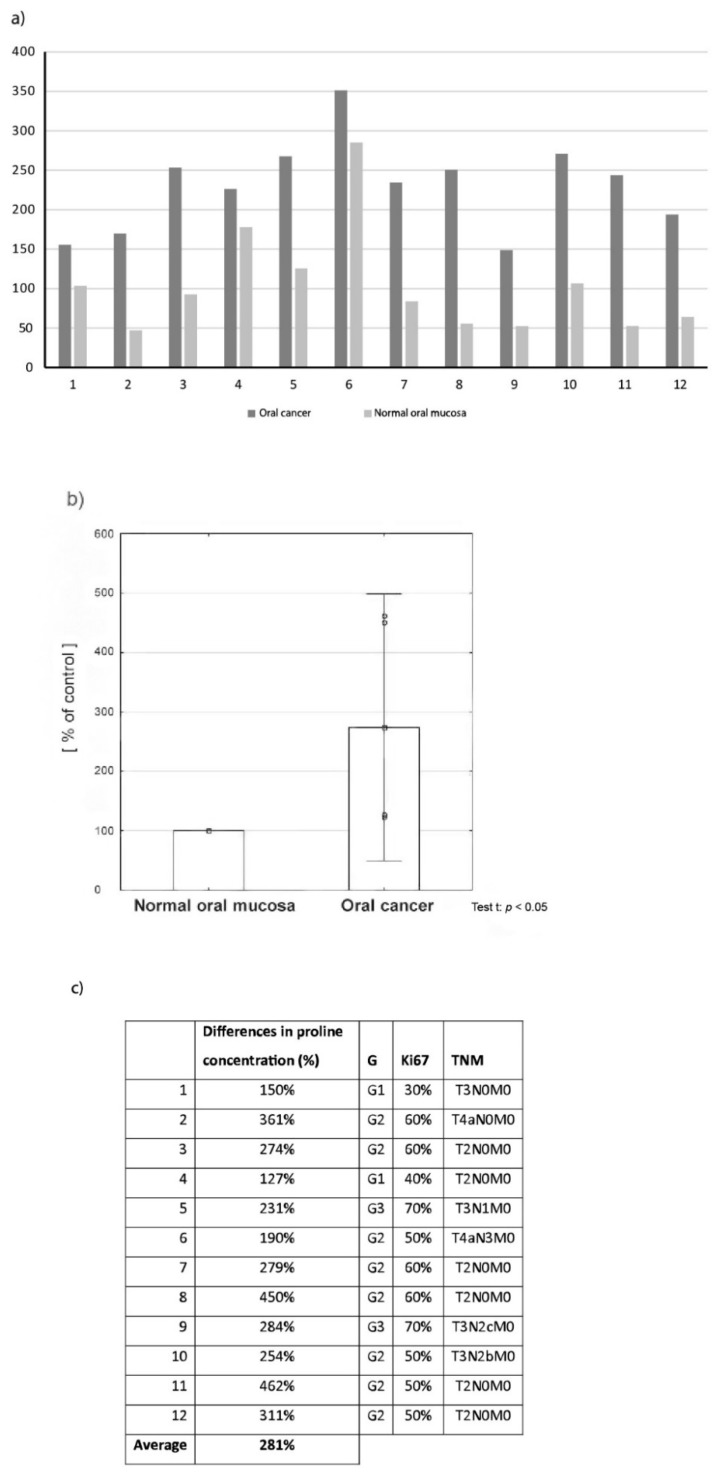
Proline concentration in oral cancer tissue in comparison to normal oral mucosa: (**a**) values presented for individual patients, (**b**) the average value of proline concentration in all cancer tissue in comparison to normal tissue, (**c**) Correlation between proline concentration in tumour tissue and tumour malignancy (histological grade and clinical stage).

Results indicate significantly higher concentration of examined amino acid in oral cancer tissue in comparison to normal tissue in all patients, values in tumour tissue ranged from 127% to 462% in reference to normal tissue (Figure 1a), the average value reached 281% (Figure 1b). In the next step the correlation between proline concentration in tumour tissue and tumour malignancy (histological grade and clinical stage) was evaluated (Figure 1c). Relatively small differences in proline content (127–150%—Cancer tissue compared to normal tissue) were found in tissue samples of highly differentiated (G1) and low TNM stage tumours. Interestingly, the biggest differences (450% and 462%) were found in the group of moderately differentiated tumours (G2) with no lymph node metastases (clinical stage T2N0M0). In the group of poorly differentiated tumours (G3), where the highest proline values were to be expected, the concentration of this amino acid in tumour tissue was 231% and 284% of the value found in normal tissue. However, these cases were advanced-stage tumours with lymph node metastases (T3N+).

### 2.2. Expression of Selected Proteins (PRODH/POX, PPAR-γ, HIF-1α) Involved in Proline Metabolism and Important in the Process of Apoptosis—Tissue Material

**Figure 2:**
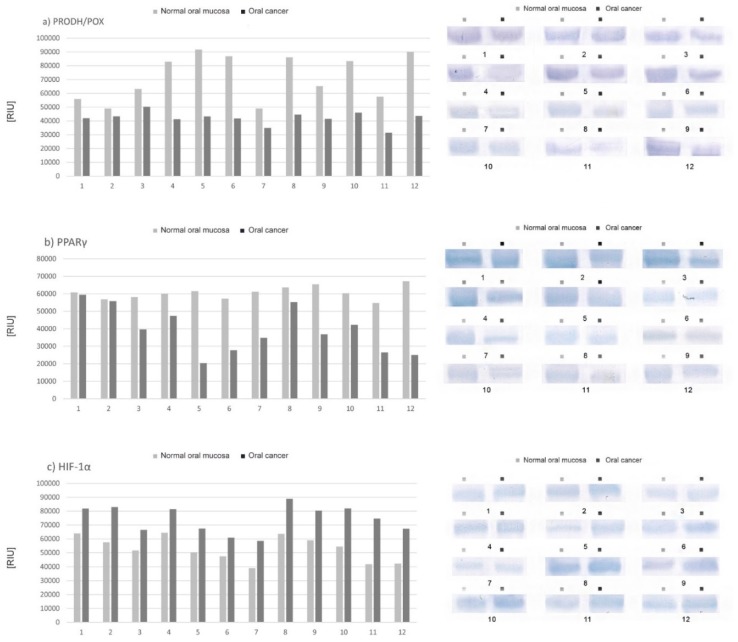
Expression of selected proteins involved in proline metabolism and important in the process of apoptosis: (**a**) proline dehydrogenase/oxidase (PRODH/POX), (**b**) PPAR-γ, (**c**) HIF-1α; the comparison of oral cancer tissue with normal oral mucosa of individual patients.

Differences in the expression of selected proteins are presented as a percentage of the value for cancer tissue compared to normal tissue (defined as 100%) in individual patients. Results indicate lower expression of PRODH/POX (Figure 2a) and peroxisome proliferator-activated receptor gamma (PPARγ) (Figure 2b) and higher expression of hypoxia-inducible transcription factor 1 alpha (HIF-1α) (Figure 2c) in oral cancer tissue in comparison to normal tissue in all participants although those differed significantly between individual patients.

### 2.3. Differences in the Expression of PRODH/POX, PPARγ and HIF-1α according to the Tumor Malignancy

**Figure 3:**
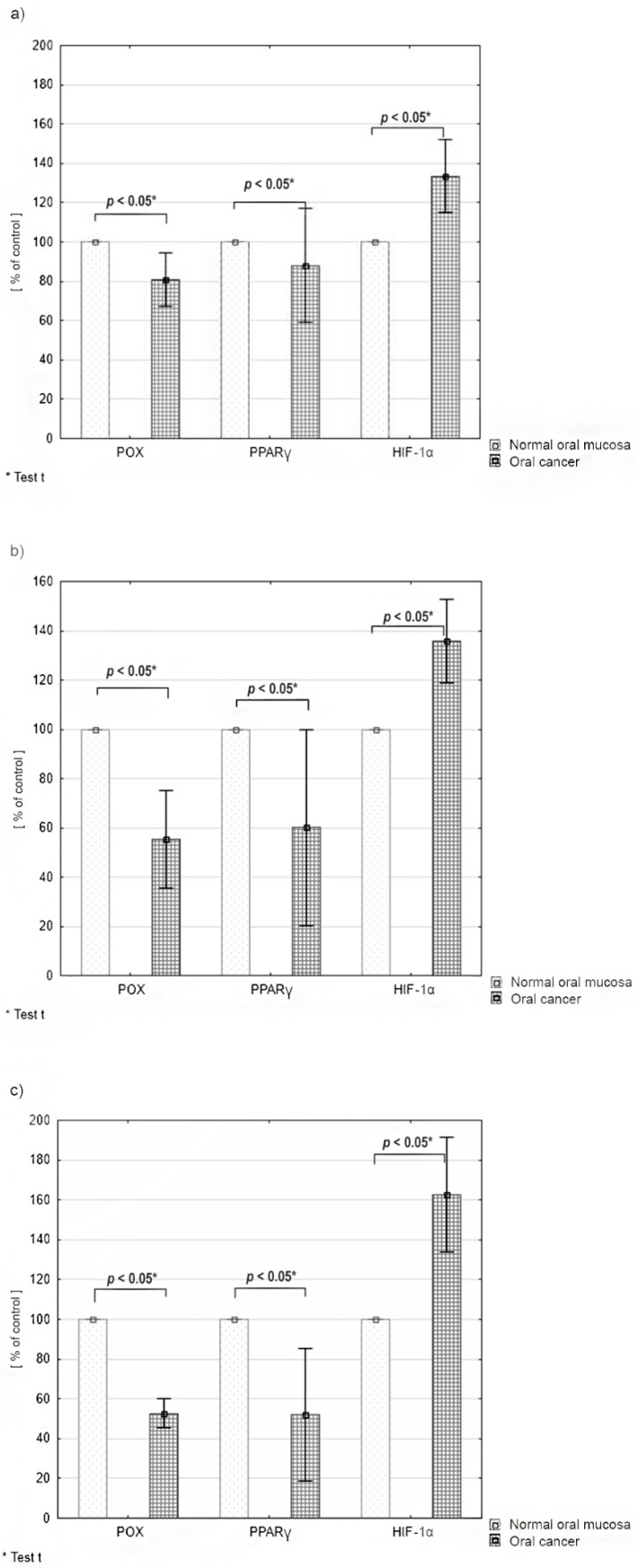
Differences in the expression of PRODH/POX, PPARγ and HIF-1α in tissue material grouped accordingly to the histological grade (G) and clinical malignancy of tumour: (**a**) G1, (**b**) G2, (**c**) G3.

Results are presented as a percentage of the mean value for cancer tissue compared to the mean value for normal tissue (defined as 100%) and arranged in groups with the same histological tumour grade (G1, G2, G3). The least distinct differences in POX, PPARγ and HIF1-α expression were found in the group of highly differentiated tumours (G1, KI-67 < 40%)—The average expression of PRODH/POX and PPARγ was 88% compared to normal tissue, while HIF1α was 130%. In this group, two cases of lymph node metastases (N2) were found, with a relatively small tumour size: T1–T2 (Figure 3a). In the group of G2 tumours (Ki-67 40–70%), differences in the expression of examined proteins reached medial values: the mean value of PRODH/POX expression in oral cancer tissue was 55%, the mean value of PPARy—60% and mean value of HIF-1α—137%. In this group, only one patient had metastases in lymph nodes and another presented with an advanced-stage tumour—T3. In other participants the clinical stage of the disease was T1-2N0M0 (Figure 3b). The most significant differences in the expression of selected proteins were observed in the group of poorly differentiated tumours (G3, KI-67 > 70%): the mean value of PRODH/POX expression was 52% compared to normal tissue, 48% of PPARγ, and 163% of HIF-1α (Figure 3c).

### 2.4. Viability Assay and Proliferation Assay Cell Culture

**Figure 4:**
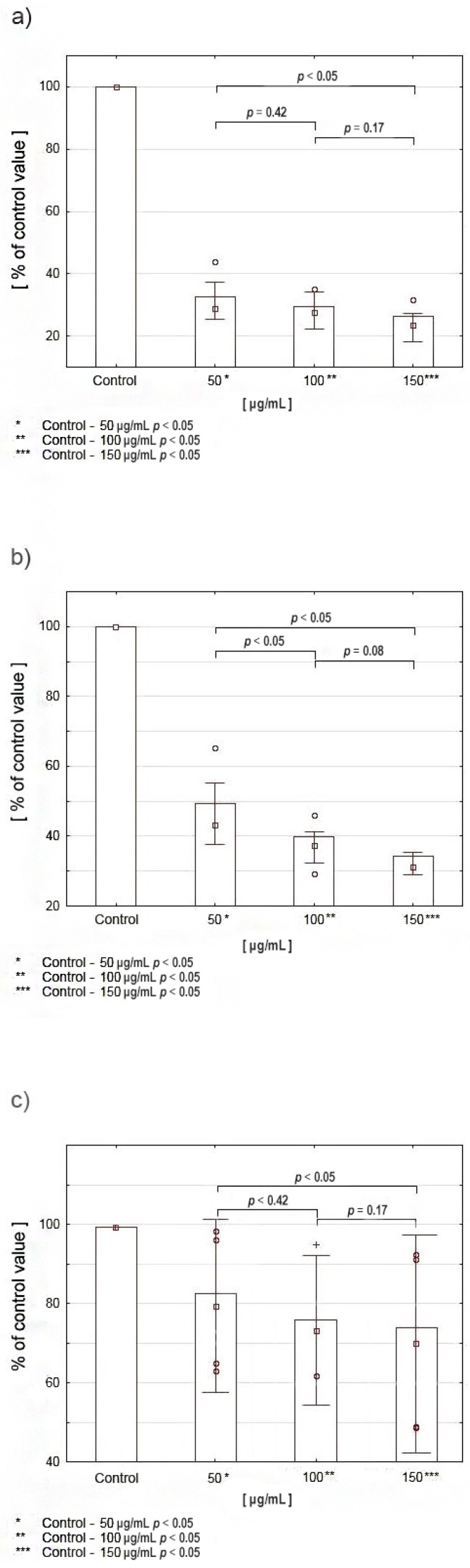
Viability assay (MTT test) in CAL-27 cells incubated with various concentrations of celecoxib: (**a**) after 24 h, (**b**) after 48 h, (**c**) after 72 h.

The results of the viability assay (MTT test) in CAL-27 cells incubated with various concentrations of celecoxib indicated a significant reduction in viability of tongue squamous cell carcinoma cells. Cell survival decreased with increasing drug concentration; values varied depending on the time of incubation and drug concentration. After 24 h, the following results were obtained: at the drug concentration 50 μg/mL cell survival reached 36% of the control value, at 100 μg/mL—35%, at 150 μg/mL—33% (Figure 4a). The results after 48 h of incubation also indicated decreased cell viability: at the concentration of 50 μg/mL the survival rate was 50% of the control value, at 100 μg/mL—40%, while at 150 μg/mL—34% (Figure 4b). After 72 h of incubation the following results were obtained: at the concentration of 50 μg/mL—82%, at 100 μg/mL—77% and at 150 μg/mL—74% (Figure 4c).

**Figure 5:**
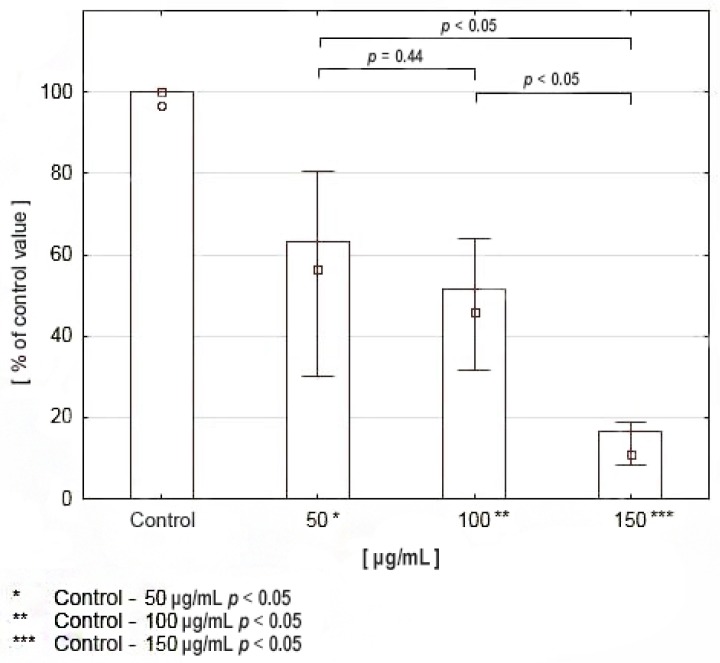
DNA biosynthesis in CAL-27 cell culture treated with various concentrations of celecoxib.

The results of the proliferation assay indicated a significant reduction in DNA biosynthesis in squamous cell carcinoma cells of the tongue compared to controls. The values differed depending on drug concentration—They decreased with increasing drug concentrations: at 50 μg/mL DNA biosynthesis reached 62% of the control value, at 100 μg/mL it reached 46%, while at 150 μg/mL it reached 19% (Figure 5).

### 2.5. Expression of PRODH/POX, PPARγ, and HIF-1α in CAL-27 Cells Incubated with Various Concentrations of Celecoxib

**Figure 6:**
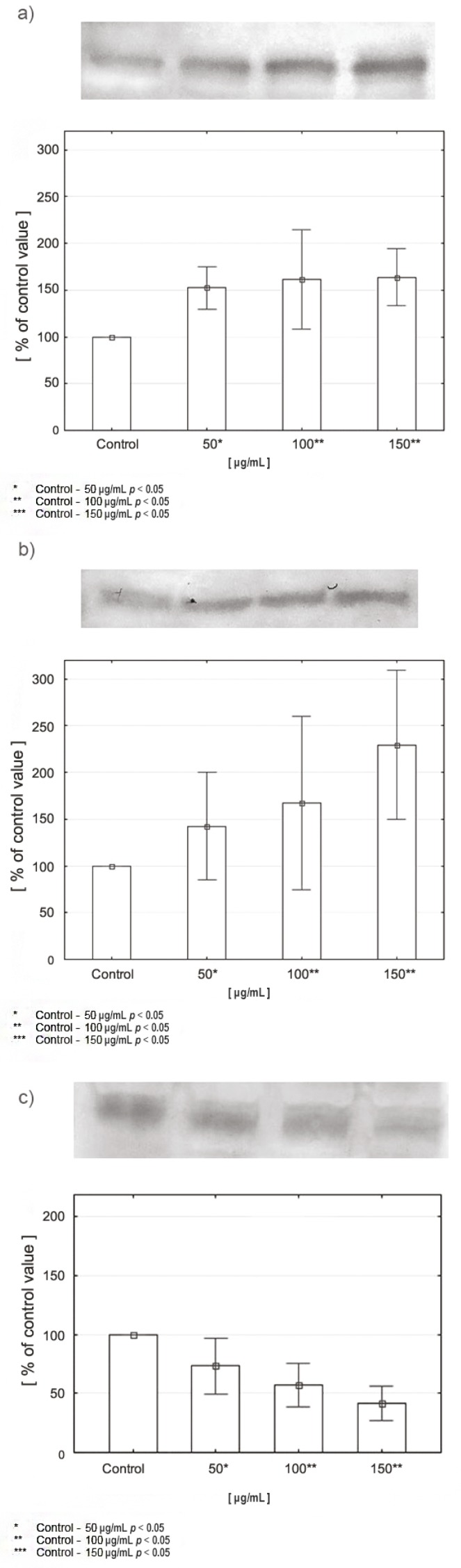
Expression of PRODH/POX (**a**), PPARγ (**b**), and HIF-1α (**c**) in CAL-27 cells incubated with various concentrations of celecoxib.

Obtained results indicated a significant increase in PRODH/POX expression in cancer cells after incubation with various concentrations of celecoxib compared to controls. Expression of this factor increases with an increase in drug concentration: at 50 μg/mL it reached 155% of the control value, at 100 μg/mL it reached 162%, while at 150 μg/mL it reached 164% (Figure 6a). The results of the test for PPARγ also indicate an increase in expression of this factor in cancer cells after incubation with celecoxib. Expression increases with increasing drug concentration: at 50 μg/mL it reached 145% compared to controls, at 100 μg/mL it reached 170%, while at 150 μg/mL it reached 230% (Figure 6b). The results of the test for HIF-1α indicate a decrease in the expression of this protein in cancer cells after incubation with celecoxib compared to controls. Expression of the selected factor decreases with increasing drug concentration: at 50 μg/mL the result reached 75%, at 100 μg/mL it reached 60%, while at 150 μg/mL it reached 40% (Figure 6c).

### 2.6. Evaluation of Apoptosis—Expression of Caspases-3 and 9 in CAL-27 Cancer Cells after Incubation with Celecoxib

**Figure 7:**
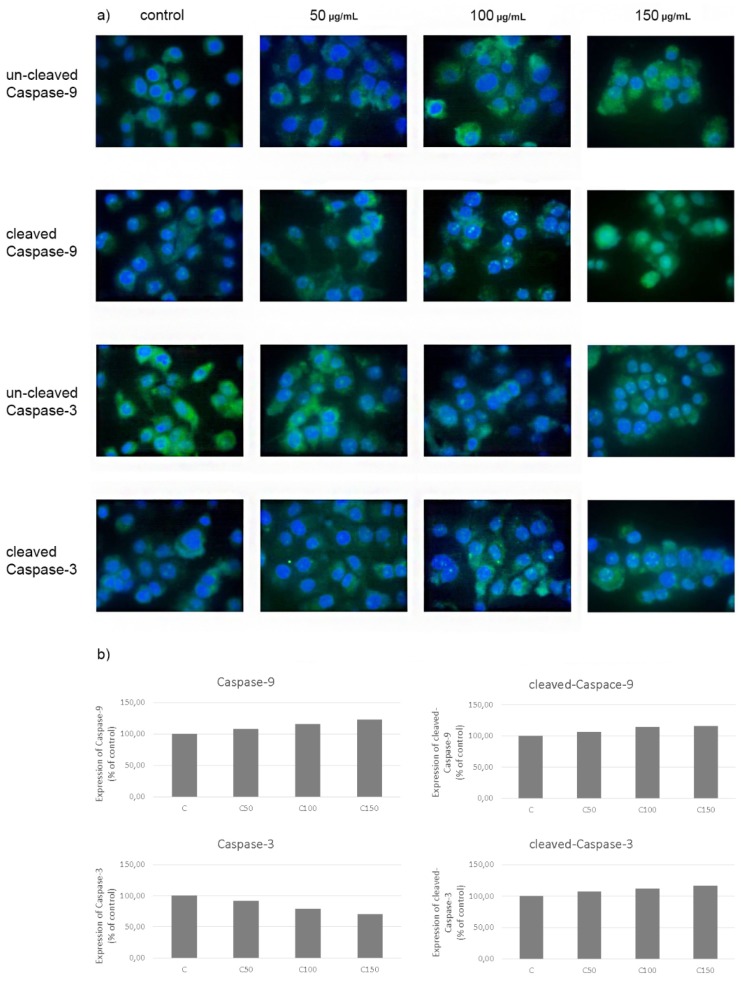
Expression of caspases-3 and 9 (cleaved and un-cleaved) in CAL-27 cancer cells after incubation with celecoxib (**a**) with quantification data (**b**).

Treatment of CAL-27 cells with celecoxib in selected concentrations upregulated the expression of cleaved and uncleaved caspase-9 and cleaved caspase-3 according to the increasing concentration of the drug. Expression of uncleaved caspase-3 decreased with the increase in drug concentration. Obtained results suggest the induction of cellular apoptosis by celecoxib (Figure 7).

## 3. Discussion

Evaluation of proline concentration in oral cancer tissue samples and normal oral mucosa harvested from patients during surgery was the first stage of our research. The obtained results indicated significantly higher proline concentration in oral cancer tissue in comparison with normal tissue in all study participants. The values differed significantly between individual patients-proline content in tumour tissue ranged from 127% to 462% in comparison to normal tissue, where the average concentration was 281% (Figure 1a,b).

Proline metabolism plays an important role not only in protein synthesis but it is also a key regulator of many biochemical processes in cells, including carcinogenesis. The majority of available studies indicate that high intracellular proline levels are associated with the development of more histologically and clinically malignant tumours, characterised by faster growth, earlier formation of metastasis in lymph nodes and shorter overall survival [11,20]. High proline levels are responsible for protecting cancer cells from oxidative stress, associated with an increase in antioxidant enzyme activity, which leads to the uptake and reduced production of reactive oxygen species [12,21,22]. Togashi et al. [11] in their research on oesophageal cancer showed that apart from decreased ROS production, high proline concentration was also associated with the overexpression of the ORAOV1 gene (Oral Cancer Overexpressed 1). Liu et al. proved that higher proline concentration in cancer cells may be associated with increased activity of the oncogenic MYC transcription factor, which contributes to an increase in proline and glutamine biosynthesis [23,24]. Increased proline levels, as one of the most important metabolic changes, have also been reported by Vermeersch et al. in their research conducted on the OVCAR-3 ovarian cancer cells [25].

Despite the fact that the majority of available studies clearly indicate that higher proline concentration in cancer cells is associated with a higher histological grade and a higher clinical stage of the tumour, we observed a diversified correlation between the concentration of the aforementioned amino acid in tumour tissue, and tumour grade and stage. Detailed results are presented in Figure 1c. However, it should be noted that since the number of patients with high (G1) and low (G3) differentiated tumours was small, there is a high probability that the results may be different in larger groups. Unfortunately, we could not harvest sufficient quantities of normal tissue for both experiments conducted on tissue material (HPLC-ESI-MS/MS and Western immunoblot) from all 24 patients as the oral cavity is a relatively small operating field and we did not want to cause additional deformities. Therefore, for each test we used tissue samples obtained from 12 patients. However, in our opinion, the presented results may provide a sound basis for further research on larger groups.

The next stage of our research was the evaluation of the expression of selected proteins (listed below) involved in proline metabolism and important in the process of apoptosis:

- PRODH/POX, pro-apoptotic enzyme (initiates apoptosis both in the intrinsic and extrinsic pathway). Its activity is regulated, among others, by PPARγ and p53- transcription factors regulating the cell cycle [13,26].
- PPARγ (peroxisome proliferator-activated receptor γ) is a ligand-activated transcription factor belonging to the superfamily of the nuclear hormone receptor. It plays an important role in regulating the process of apoptosis [27,28].
- HIF-1α (hypoxia-inducible factor 1-alpha) is a transcription factor specifically activated by low oxygen concentration in the cellular environment. It is an indispensable component of the transcriptional response of tumours to hypoxia. It also influences an increase in the expression of key factors determining cancer development, e.g., VEGF, COX-2, NF-κB [10,29,30].

The results of our study demonstrated lower expression of PRODH/POX and PPARγ and higher expression of HIF-1α in oral cancer tissue in comparison to normal tissue in all study participants, although the obtained values differed significantly between individual patients. Furthermore, significant correlations were found between the expression of individual proteins and the histological differentiation of the tumour, and, partly, the clinical stage of the disease (Figure 2).

The least distinct differences in POX, PPARγ and HIF1-α expression occurred in the group of highly differentiated tumours (G1). In the group of G2 tumours, differences in the expression of the examined proteins reached medial values, while the most significant differences in the expression of the selected proteins were observed in the group of poorly differentiated tumours (G3).

Considering the results of the tests conducted on tissue material, as well as the fact that PRODH/POX, PPARγ activation and HIF-1α inhibition is associated with the activation of apoptosis and inhibition of tumour growth, we decided to use a cell culture model to examine how the aforementioned parameters changed after treatment. We used celecoxib as a drug regulating these pathways. It is an anti-inflammatory drug, a selective cyclooxygenase II (COX-2) inhibitor. However, it has also been proven to act on a number of other factors, frequently related to COX-2, which, inter alia, affect cancer cell growth. Studies evaluating the anticancer potential of celecoxib have been performed on cells and tissues of various types of cancer. They have demonstrated a significant inhibitory effect of celecoxib on the development of major malignancies in humans including breast, prostate, colon, lung as well as other tumours, such as ovarian, head and neck, pancreatic, and stomach cancer [17,18,31,32,33]. However, its mechanism of action in this area has not been fully elucidated. Interestingly, it has been indicated in the literature that there is a possibility of cross-talk between COX-2 and PRODH/POX pathways [34]. Considering the relationships mentioned above, we assumed that celecoxib, as an inhibitor of the COX-2 pathway, could be a potential PRODH/POX activator. We have not found any reports on the impact of this drug on proline dehydrogenase/oxidase or on apoptosis initiated by this pathway.

The results of the viability assay (MTT test) assessed after 24, 48 and 72 h of incubation with various concentrations of celecoxib indicated a significant reduction in the viability of squamous cell carcinoma cells. Cell survival decreased with increasing drug concentration, the values varied depending on the duration of incubation and drug concentration. The results are presented in Figure 4. In order to assess whether celecoxib also affected cell proliferation, the evaluation of DNA biosynthesis was performed. The results indicated a significant reduction in DNA biosynthesis in CAL-27 cells compared to controls. The values differed depending on drug concentration. Results are presented in Figure 5.

Other preclinical studies involving the use of celecoxib at similar doses have demonstrated the drug’s ability to hinder the proliferation of tumour cells. In addition, cell cycle arrest has also been observed as well as the induction of apoptosis and inhibition of angiogenesis in cell cultures and animal models of colon, breast, prostate, nasopharyngeal and lung cancer [35,36,37,38].

The results of the Western immunoblot test performed on tissue material revealed significant differences in the expression of selected proteins involved in apoptosis-related proline metabolism (PRODH/POX, PPARγ, HIF-1α) between squamous cell carcinoma and normal mucosa. To determine changes in the expression of these factors under the influence of celecoxib, the same test was performed on CAL-27 cells after 24 h of incubation with selected concentrations of the drug.

The results indicated a significant increase in PRODH/POX and PPARγ expression in cancer cells treated with various concentrations of celecoxib compared to controls. Both factors were upregulated in accordance with the increase in drug concentration. In contrast to previous results, the expression of HIF-1α was significantly decreased—It increased inversely to drug concentration (Figure 6).

Various mechanisms of the anticancer potential of celecoxib have been indicated in the available literature. Many authors suggest that it is not only the inhibition of COX-2 but the regulation of other factors involved in the development of malignant tumours by the drug that has a considerable impact on hindering tumour growth (i.e., a decrease in OCT4, SOX2, and BMP7 gene expression, increase in the expression of E-cadherin gene, Wnt/β-catenin, cell adhesion molecules, and surface receptors: AMFR and EGFR, induction of p53 gene expression) [39]. However, no evidence of the drug’s effect on PRODH/POX activity has been demonstrated to date. Only Liu et al. showed a correlation between PRODH/POX and COX-2, where an increase in PRODH/POX activity was associated with the downregulation of COX-2. Studies conducted in cell culture as well as animal models of colorectal cancer have shown that the inhibition of the COX-2/PGE2 pathway plays an important role in apoptosis induced by the PRODH/POX pathway. It has also been proven that the inhibition of these factors by PRODH/POX is caused by increased production of reactive oxygen species [34].

Having considered the effects described above, we assumed that celecoxib as a PRODH/POX activator can promote apoptosis through this pathway (both intrinsic and extrinsic). By generating reactive oxygen species it can induce caspase-9 activity, which is one of the stages of mitochondrial apoptosis (intrinsic pathway). PRODH/POX can also stimulate the expression of TRAIL and DR5, which stimulate the extrinsic apoptotic pathway [13,26]. Some studies have indicated the role of PRODH/POX as a further effector of apoptosis activation associated with p53 and PPARγ proteins—factors responsible for regulating the cell cycle, inhibitors of tumorigenesis [10,13,14,26,40,41].

To clarify the effect of celecoxib on apoptosis initiated by the PRODH/POX pathway, we decided to evaluate apoptosis activation in CAL-27 cells incubated with celecoxib. The essential mediators of apoptotic cell death are caspases. For this purpose, the expression of caspases-3 and -9 (cleaved and uncleaved) was analysed using immunostaining and confocal microscopy.

Caspase-9 is activated during intrinsic apoptosis. The process starts with the permeabilisation of the mitochondrial outer membrane, resulting in Cytochrome c activation and apoptosome formation. Subsequently, caspase-9 is activated and it can directly cleave and activate caspase-3 and caspase-7 [3,42,43]. Caspase-3 is active in the executive phase of apoptosis. It is responsible for the structural and enzymatic damage of proteins, leading to chromatin condensation, DNA fragmentation and total cell disintegration [44,45].

The results of our research indicate that the treatment of CAL-27 cells with celecoxib upregulated the expression of cleaved and uncleaved caspase-9 and cleaved caspase-3. The expression of uncleaved caspase-3 decreased with an increase in the drug’s concentration (Figure 7). The increase in caspase-9 expression (cleaved and uncleaved) indicates that celecoxib stimulates the activation of this protein and, as a result, promotes the induction of apoptosis. Changes in the quantity of the uncleaved and cleaved forms of caspase-3 observed in cells treated with celecoxib indicate the activation of this protein by active caspase-9. The increased amount of the cleaved form of caspase -3 is associated with the executive phase, the final stage of apoptosis.

To the best of our knowledge, this is the first study demonstrating apoptosis activation by celecoxib via the PRODH/POX pathway in cancer cells.

## 4. Materials and Methods

### 4.1. Patients and Tissues

Two types of tissue were studied: histopathologically confirmed oral squamous cell carcinoma tissue samples (irrespective of location) and clinically unchanged mucosa of the oral cavity. Twenty four patients treated in the Department of Maxillofacial Surgery in Bialystok were enrolled in the study, regardless of age, gender and type of treatment received. To evaluate the clinical stage of the disease, the TNM classification was used [46]. Moreover, as an indicator of histological malignancy, the histopathological grade of the tumour (G1-G4) was determined [47] as well as the nuclear expression of the KI-67 protein (the highest percentage of cells with nuclear KI-67 expression in 10 fields of view at 200× magnification). All patients provided written informed consent prior to the harvesting of tissue samples. The study obtained the approval of the Bioethics Committee of the Medical University of Bialystok (R-I-002/385/2016). The participants’ identities remained anonymous (according to the Declaration of Helsinki).

### 4.2. Evaluation of Proline Concentration in Tissue Material—Liquid Chromatography Combined with Tandem Mass Spectrometry (HPLC-MS/MS)

To all tissue samples weighing 50 mg, 200 μL of methanol and the same amount of water (MiliQ) were added in a 1:1 ratio. Then, the tissues were homogenised and proline concentration was determined using the combination of the aTRAQ™ Kit for amino acid analysis (SCIEX, Framingham, MA, USA) and LC-MS/MS analysis. Samples for the aTRAQ™ Kit were prepared in accordance with the manufacturer’s protocol. LC-MS/MS analysis was performed using a 1260 Infinity HPLC instrument (Agilent Technologies, Santa Clara, CA, USA) and a 4000 QTRAP mass spectrometer (SCIEX, Framingham, MA, USA) equipped with an electrospray ionisation source. Sample analyses were performed in a random order. Detailed specifications of sample preparation and LC-MS/MS parameters have been described in our previous publications [48,49]. Data acquisition and management were conducted using Analyst 1.5.2 software (Sciex, Framingham, MA, USA).

### 4.3. Expression of Selected Proteins by Western Immunoblot—Tissues

TRIS-HCl (0.05 mol/L pH 7.6, 20% *w*/*v*) was added to tissue samples of the same weight (50 mg). The samples were then homogenised and centrifuged (16,000× *g*, 30 min, 4 °C). The amount of the obtained supernatants with identical protein content (25 μg) was analysed. Protein content was evaluated using the method of Lowry et al. [50]. Next, SDS-PAGE electrophoresis of proteins was performed according to the Laemli method [51]. Then, the proteins were transferred to the nitrocellulose membrane (semi-dry transfer) with the help of the TransBlot^®^SD Cell electrotransfer kit. Subsequently, immunodetection of specific proteins was performed using the SNAP i.d 2.0 Protein Detection System (Sigma, Merck Millipore, Burlington, MA, USA). The following antibodies were used for the study: goat monoclonal anti-PRODH/POX antibody (Everest Biotech, Upper Heyford, UK), mouse monoclonal anti-PPARγ antibody (Santa Cruz, Biotechnology, CA, USA), mouse monoclonal anti-HIF-1α antibody (BD Biosciences, San Jose, CA, USA), secondary polyclonal anti-goat and anti-mouse antibodies labelled with alkaline phosphatase (Sigma-Aldrich, Corp, United States). Following the sequence of incubation with primary and secondary antibodies, the membranes were rinsed with the Sigma-Fast BCIP/NBT detection reagent (Sigma-Aldrich, St. Louis, MO, USA) to stain the immunoreactive protein. Band intensity was quantified with the densitometric method using the ImageJ software (NIH, Bethesda, MD, USA).

The results were presented as differences in the intensity of units (RIU) between oral cancer tissue and normal oral mucosa. Considering the differences observed between individual patients, we decided to compare the results with the histological grade and clinical stage of the tumour in each patient. For this purpose, the patients were divided into three groups (G1, G2, G3)—The main criterion for patient classification was the histological grade of the tumour (G), although the KI-67 index and TNM classification were also taken into consideration when grouping patients. Differences in the expression of selected proteins were presented as a percentage of the value for cancer tissue compared to normal tissue, defined as 100% (mean values for the examined tissue types in groups with the same histological tumour grade). All original datea of Western blot are available in Supplementary File S1.

### 4.4. Cell Culture

The cell line of squamous cell carcinoma of the tongue was used in the study (CAL-27, CRL-2095 TM, ATCC, Manassas, VA, USA). As a growth medium, DMEM was used, supplemented with 10% FBS, 50 U/mL penicillin, 50 μg/mL streptomycin and 2 mmol/L glutamine. The cell culture was incubated at 37 °C and in 5% CO_2_. After approximately 70% of confluency was obtained, celecoxib (Sigma-Aldrich, St. Louis, MO, USA) was added to the cells at the concentrations of 50, 100, 150 μg/mL. Then, the cells were incubated for 24 h under the same conditions. After this period of time, the cells were used in the experiments.

### 4.5. Cell Viability Assay—MTT Test

The evaluation of celecoxib cytotoxicity on tongue cancer cells was performed using methyl thiazolyl tetrazolium (MTT) in accordance with the Carmichael method [52]. Cells were cultured in standard conditions. Incubation with celecoxib at selected concentrations was continued for 24, 48 and 72 h. After that, absorbance was measured using the Asys UVM 340 microplate reader (Biogenet) at 570 nm. The results were presented as a number of viable cells expressed as a percentage of the values obtained for the control group.

### 4.6. Proliferation Assay—DNA Biosynthesis

The radiometric method was used to determine proliferation of the examined cells, assessing the incorporation of [methyl-^3^H] thymidine (Hartman Analytic GmbH, Brunswick, Germany) into cell DNA, following the application of celecoxib at various concentrations. The cells were cultured in previously described conditions. Incubation with celecoxib was continued for 24 h. Radioactivity was measured using the Tri-Carb 2810 TR Scintillation Analyzer (Perkin-Elmer, Waltham, MA, USA).

### 4.7. Expression of Selected Proteins by Western Immunoblot—Cell Lines

Evaluation of the expression of selected proteins involved in proline metabolism (PRODH/POX, PPARγ, HIF-1α) in CAL-27 cells was conducted using the Western Immunoblot method according to the same protocol as that followed during the performance of tissue material assays.

### 4.8. Evaluation of Apoptosis—Immunofluorescence Microscopy

Cells grown on a coverslip were fixed with 3.7% paraformaldehyde and permeabilised with 0.01% Triton. After blocking with 3% FBS, the cells were incubated with primary antibodies (caspase-3, cleaved-caspase-3, caspase-9, cleaved-caspase-9) and subsequently, with a FITC Fluor-conjugated secondary antibody. The cells were washed with PBS and incubated with Hoechst 33342. Samples were visualised with a confocal laser scanning microscope (BD Pathway 855 Bioimager, enlargement—40×) using AttoVision software.

### 4.9. Statistical Analysis

Statistical analysis was performed using Statistica 13 software (StatSoft). The presented results are mean values of at least six measurement points ± standard error of the mean (SEM). The one-way ANOVA analysis and the Tukey test were used to evaluate statistical significance. Values for which *p* < 0.05 were considered statistically significant.

## 5. Conclusions

The results of the presented research indicate altered proline metabolism in oral cancer tissue in comparison to normal tissue. We are the first researchers to demonstrate that celecoxib affects proline metabolism, causes the induction of apoptosis through increased expression of pro-apoptotic factors (PRODH/POX and PPARγ) and reduction of HIF-1α, which indicates the potential of this drug to inhibit tumour growth, tumour invasion and angiogenesis. Due to its role in the process of apoptosis, PRODH/POX can be of great significance in anticancer therapy. The presented results provide a basis for further research necessary to elucidate other aspects of the changes occurring in oral cancer cells under the influence of celecoxib and to assess the efficacy of celecoxib in vivo.

## Supplementary Materials

The following are available online at https://www.mdpi.com/2072-6694/12/1/136/s1, File 1: Figure S1: Expression of PRODH/POX, PPARγ and HIF-1α in tissues of patients―oral cancer (C) and healthy oral mucosa (H). Table S1: Densitometry of the Western blot figures showing the expression of PRODH/POX, PPARγ and HIF-1α in tissues of patients. Figure S2: Expression of PRODH/POX, PPARγ and HIF-1α in OSCC cancer cell lines (CAL-27) after incubation with selected concentrations of celecoxib. Table S2: Densitometry of the Western blot figures showing the expression of PRODH/POX, PPARγ and HIF-1α in OSCC cancer cell lines (CAL-27) after incubation with celecoxib.
