# Supplementary material for: Proline-Dependent Induction of Apoptosis in Oral Squamous Cell Carcinoma (OSCC)—The Effect of Celecoxib
Source: Cancers (Basel). 2020 Jan 6;12(1):136. doi: 10.3390/cancers12010136 (PMC7016823; doi:10.3390/cancers12010136)
Supplement: Supplementary file 1 [file cancers-12-00136-s001.pdf]

Figure S1

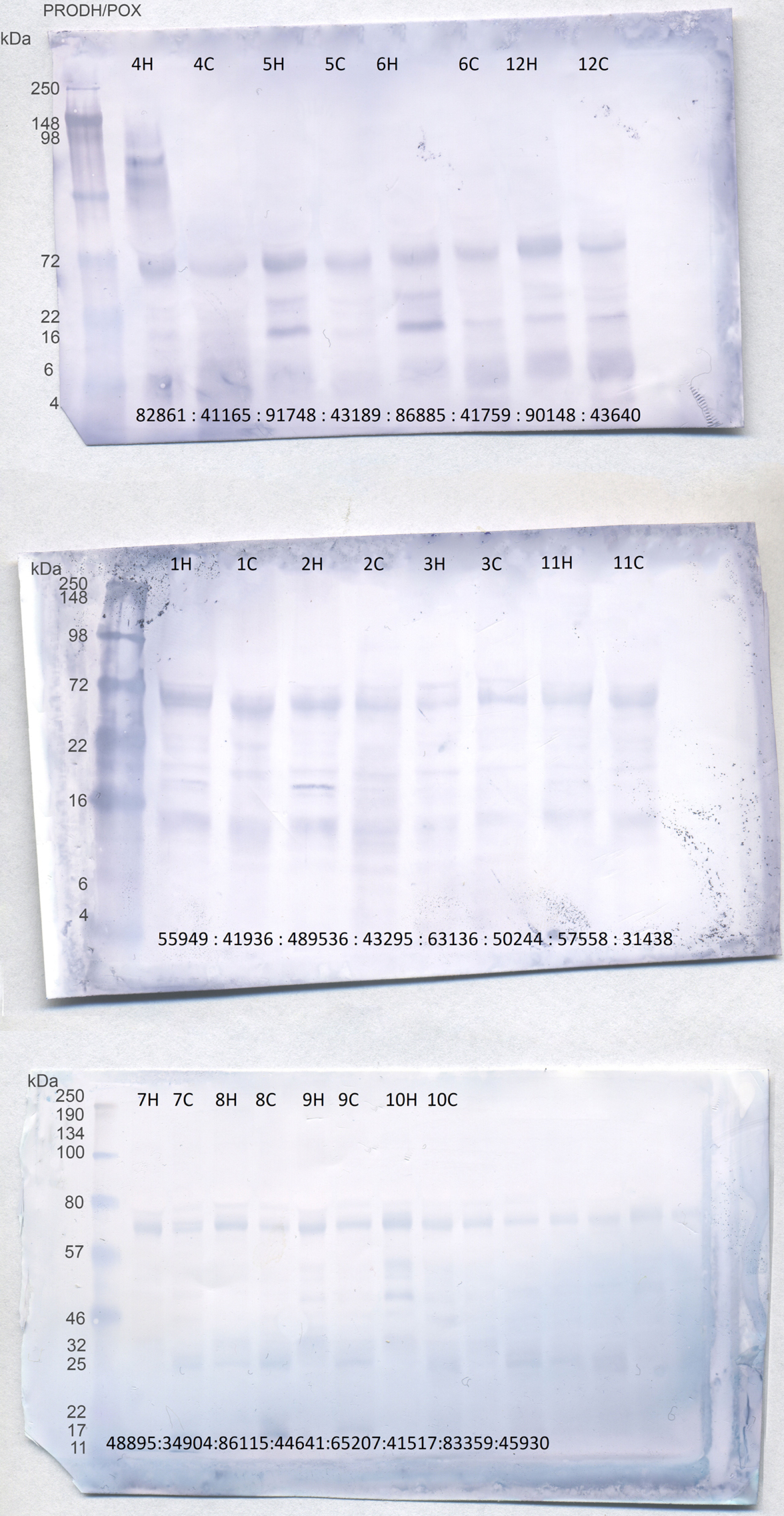
PRODH/POX
kDa
4H 4C 5H 5C 6H 6C 12H 12C
250
148
98
72
22
16
6
4
82861 : 41165 : 91748 : 43189 : 86885 : 41759 : 90148 : 43640
kDa
1H 1C 2H 2C 3H 3C 11H 11C
250
148
98
72
22
16
6
4
55949 : 41936 : 489536 : 43295 : 63136 : 50244 : 57558 : 31438
kDa
7H 7C 8H 8C 9H 9C 10H 10C
250
190
134
100
80
57
46
32
25
22
17
11
48895:34904:86115:44641:65207:41517:83359:45930

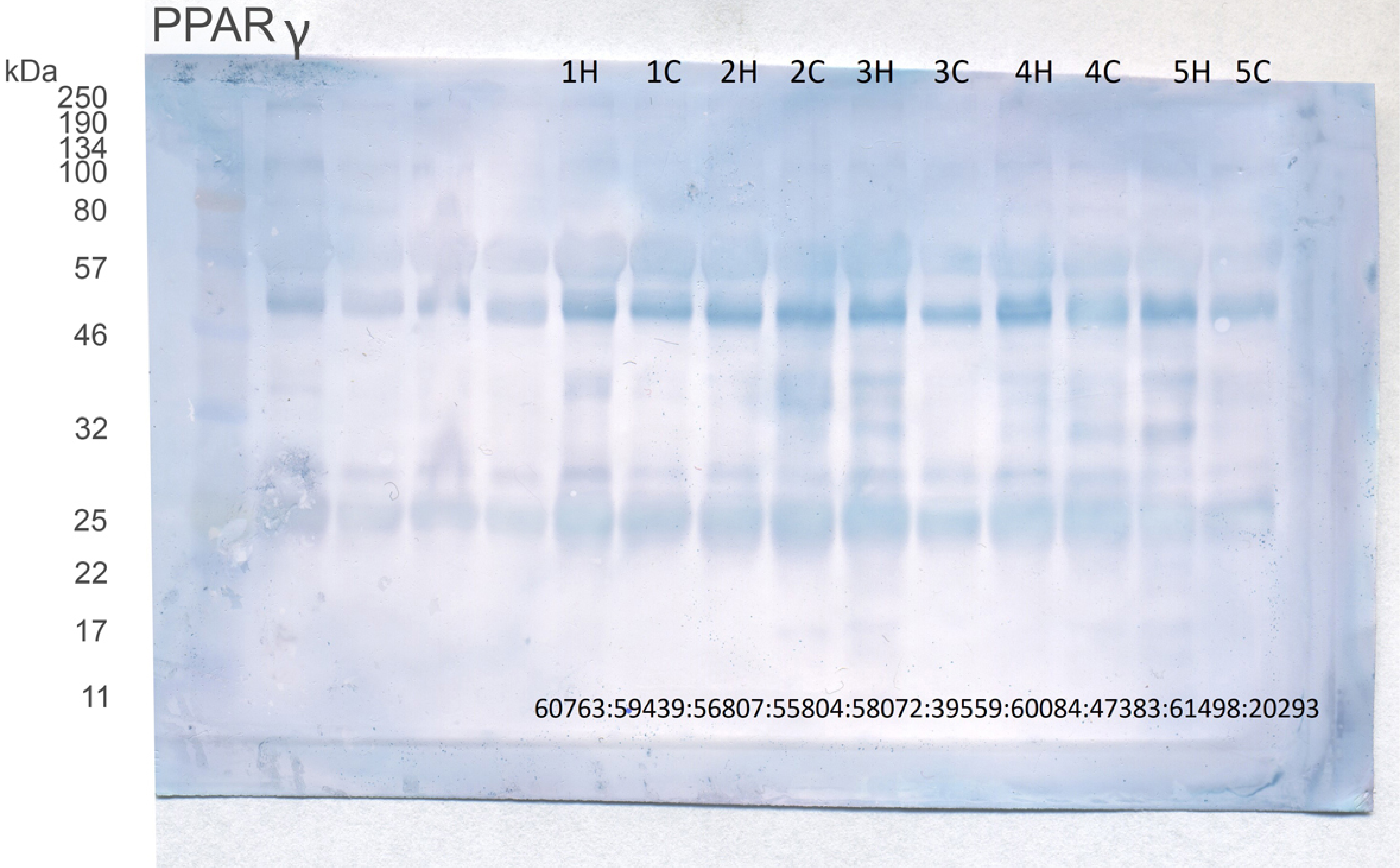
PPARγ
kDa
250
190
134
100
80
57
46
32
25
22
17
11
1H 1C 2H 2C 3H 3C 4H 4C 5H 5C
60763:59439:56807:55804:58072:39559:60084:47383:61498:20293

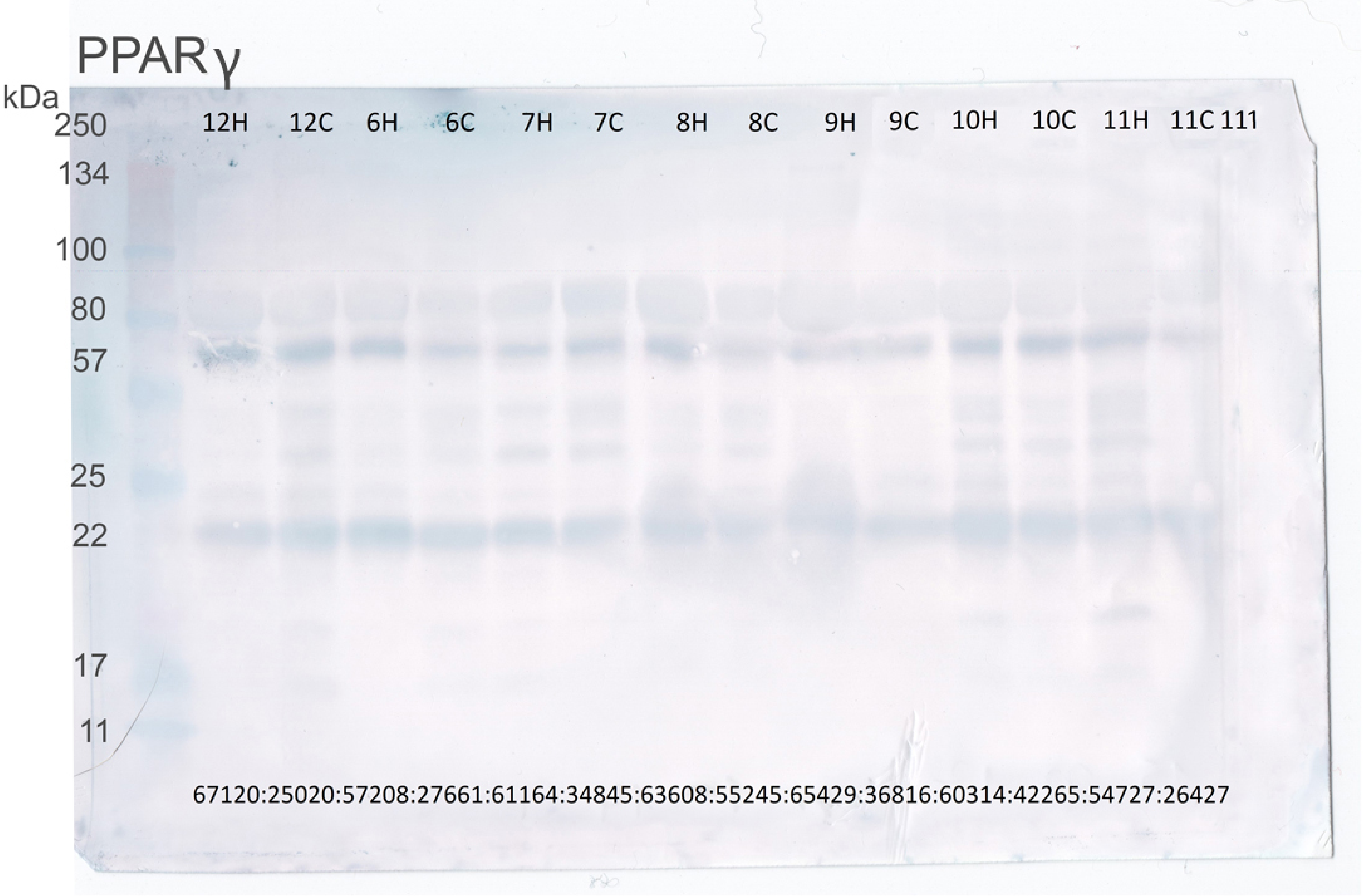
PPARγ
kDa
250
134
100
80
57
25
22
17
11
12H 12C 6H 6C 7H 7C 8H 8C 9H 9C 10H 10C 11H 11C 111
67120:25020:57208:27661:61164:34845:63608:55245:65429:36816:60314:42265:54727:26427

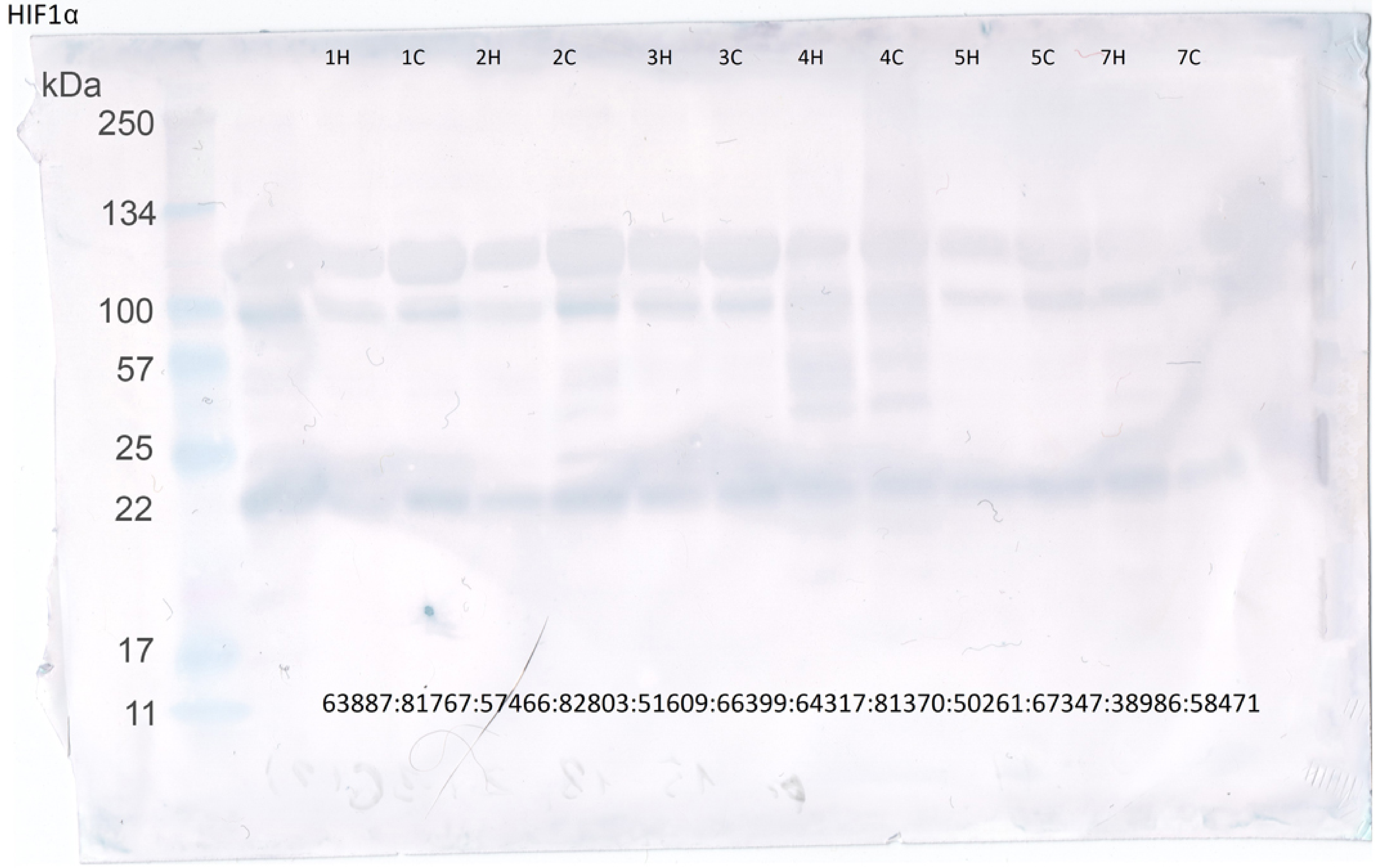
HIF1α
kDa
250
134
100
57
25
22
17
11
1H 1C 2H 2C 3H 3C 4H 4C 5H 5C 7H 7C
63887:81767:57466:82803:51609:66399:64317:81370:50261:67347:38986:58471

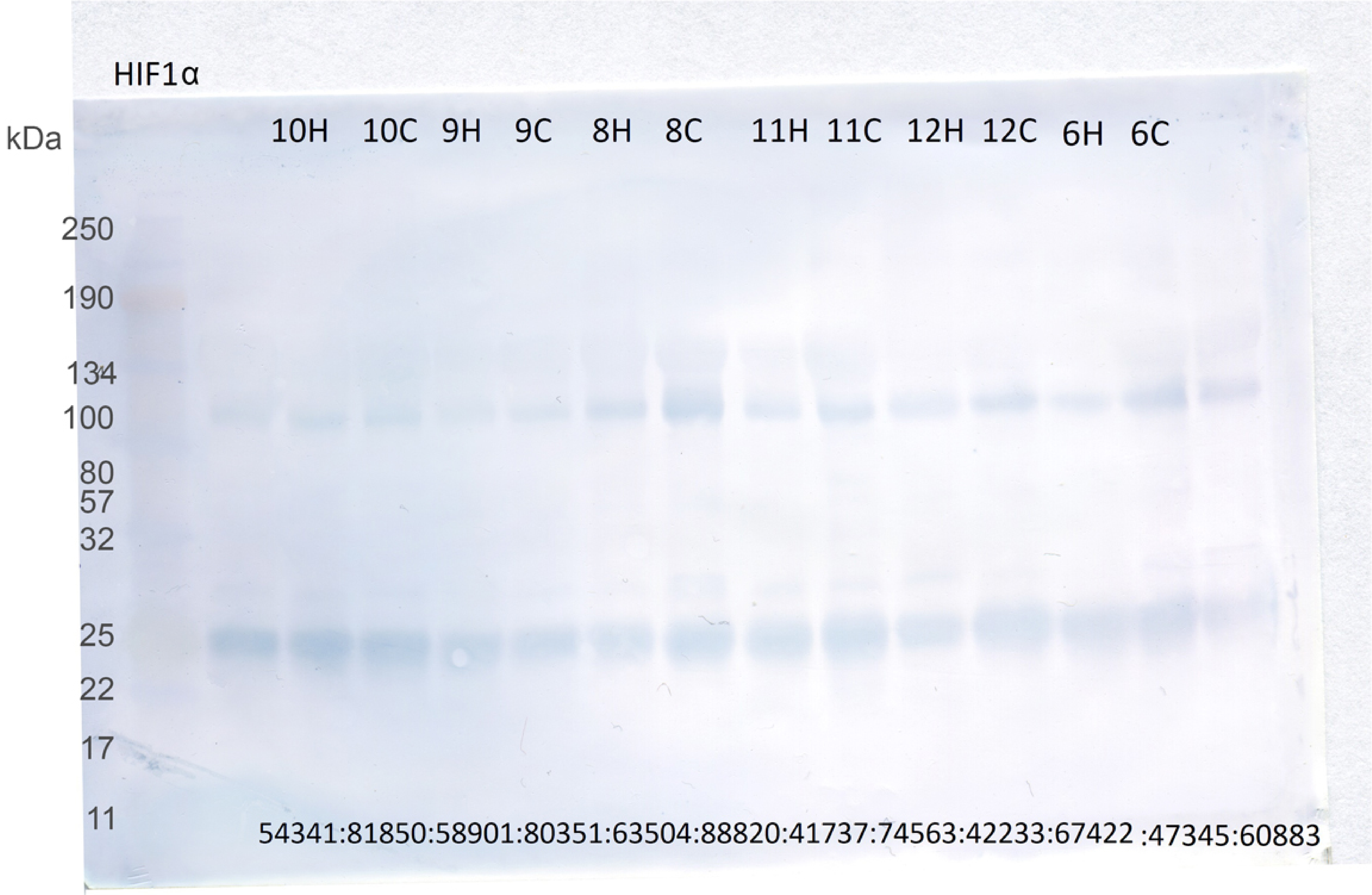
HIF1α
kDa
10H 10C 9H 9C 8H 8C 11H 11C 12H 12C 6H 6C
250
190
134
100
80
57
32
25
22
17
11
54341:81850:58901:80351:63504:88820:41737:74563:42233:67422 :47345:60883

Table S1

		Densitometry		
Number	Tissue	**POX**	**PPARy**	**HIF1a**
1	Healty	55949,4	60763,2	63886,9
	Cancer	41936,3	59438,7	81766,6
2	H	48953,5	56806,7	57465,6
	C	43294,6	55803,8	82804,7
3	H	63135,7	58072,1	51608,5
	C	50243,9	39558,8	66398,6
4	H	82861,2	60083,8	64317,3
	C	41164,8	47382,6	81370,4
5	H	91748,3	61497,7	50260,6
	C	43189,3	20292,5	67347,1
6	H	86884,8	57208,4	47345,4
	C	41759	27661,3	60882,6
7	H	48895	61163,7	38985,9
	C	34903,9	34844,7	58470,6
8	H	86115,2	63608,2	63504
	C	44641,2	55244,5	88820
9	H	65207,2	65429,3	58900,9
	C	41517,1	36815,5	80350,5
10	H	83358,5	60314	54340,7
	C	45930,2	42265,2	81849,7
11	H	57557,6	54727,1	41737,2
	C	31438,4	26426,8	74563,3
12	H	90147,7	67119,9	42232,6
	C	43639,7	25020,1	67422,1

Figure S2

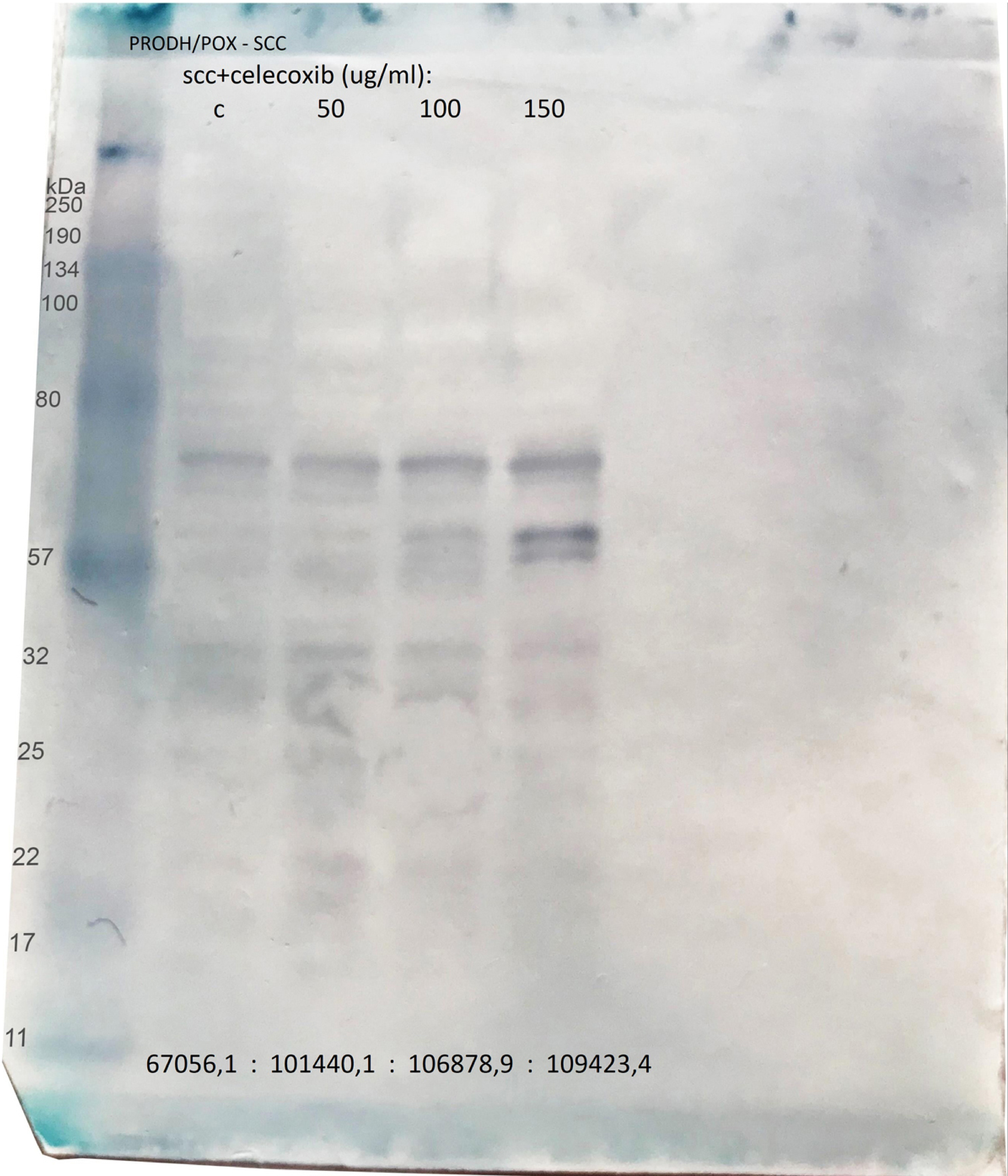

PRODH/POX - SCC
scc+celecoxib (ug/ml):
c
50
100
150
kDa
250
190
134
100
80
57
32
25
22
17
11
67056,1 : 101440,1 : 106878,9 : 109423,4

PPR- SCC

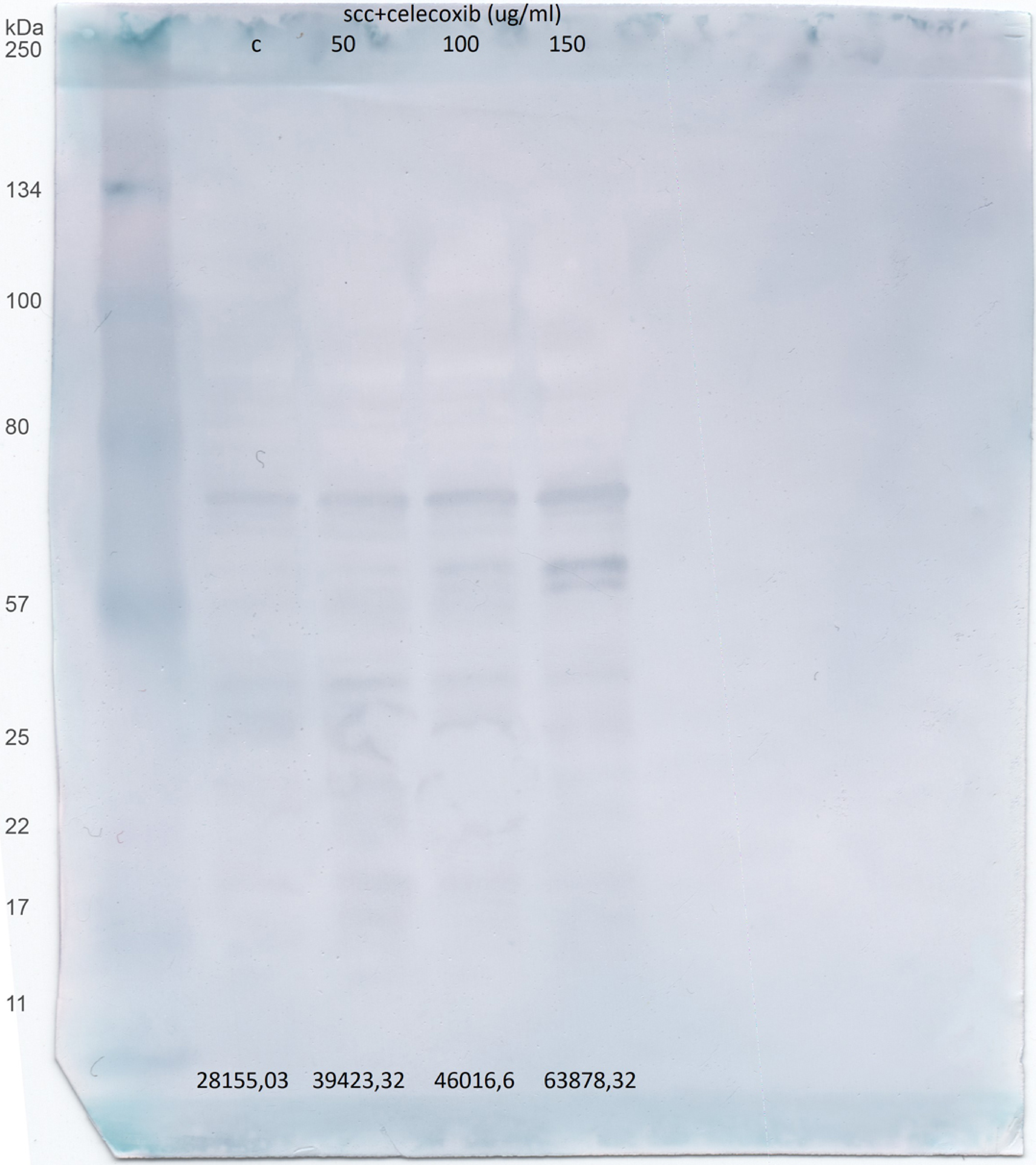
scc+celecoxib (ug/ml)
c
50
100
150
kDa
250
134
100
80
57
25
22
17
11
28155,03
39423,32
46016,6
63878,32

HIF1A-SCC

kDa
250
148
98
72
22
16
6
4

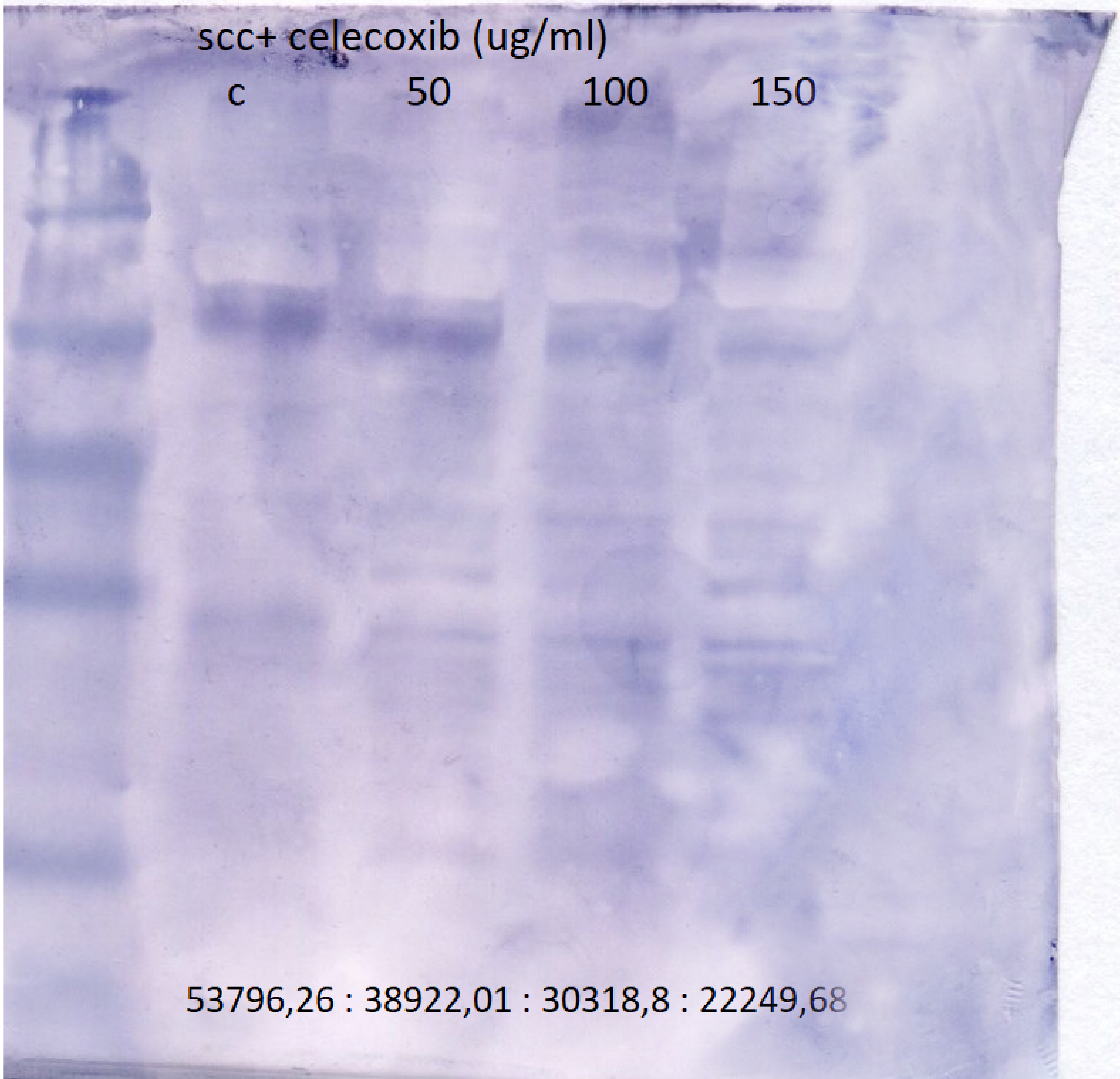

Table S2

	Densitometry - SCC+ celecoxib		
	POX	PPARy	HIF1a
C	67056,104	28155,0313	53796,259
50	101440,06	39423,321	38922,007
100	106878,89	46016,6043	30318,797
150	109423,39	63878,3235	22249,68
